# Preexisting hypertension and pregnancy-induced hypertension reveal molecular differences in placental proteome in rodents

**DOI:** 10.1152/physiolgenomics.00160.2020

**Published:** 2021-05-10

**Authors:** Sheon Mary, Heather Small, Florian Herse, Emma Carrick, Arun Flynn, William Mullen, Ralf Dechend, Christian Delles

**Affiliations:** ^1^Institute of Cardiovascular and Medical Sciences, University of Glasgow, Scotland, United Kingdom; ^2^Experimental and Clinical Research Center, a joint cooperation between Max-Delbrück-Center for Molecular Medicine and Charité-Universitätsmedizin Berlin, Berlin, Germany; ^3^Department of Cardiology and Nephrology, HELIOS Clinic, Berlin, Germany

**Keywords:** chronic hypertension, hypertensive pregnancy, label-free proteomics, placental biology, preeclampsia rat model

## Abstract

Preexisting or new onset of hypertension affects pregnancy and is one of the leading causes of maternal and fetal morbidity and mortality. In certain cases, it also leads to long-term maternal cardiovascular complications. The placenta is a key player in the pathogenesis of complicated hypertensive pregnancies, however the pathomechanisms leading to an abnormal placenta are poorly understood. In this study, we compared the placental proteome of two pregnant hypertensive models with their corresponding normotensive controls: a preexisting hypertension pregnancy model (stroke-prone spontaneously hypertensive rats; SHRSP) versus Wistar–Kyoto and the transgenic RAS activated gestational hypertension model (transgenic for human angiotensinogen Sprague-Dawley rats; SD-PE) versus Sprague-Dawley rats, respectively. Label-free proteomics using nano LC-MS/MS was performed for identification and quantification of proteins. Between the two models, we found widespread differences in the expression of placental proteins including those related to hypertension, inflammation, and trophoblast invasion, whereas pathways such as regulation of serine endopeptidase activity, tissue injury response, coagulation, and complement activation were enriched in both models. We present for the first time the placental proteome of SHRSP and SD-PE and provide insight into the molecular make-up of models of hypertensive pregnancy. Our study informs future research into specific preeclampsia and chronic hypertension pregnancy mechanisms and translation of rodent data to the clinic.

## INTRODUCTION

Hypertension is the common medical disorder of pregnancy that complicates up to 10% of pregnancies. Hypertensive disorders can be classified into four groups: chronic hypertension, preeclampsia, superimposed preeclampsia upon chronic hypertension, and gestational hypertension (1). Preeclampsia remains the leading cause of fetal and maternal morbidity and mortality that occurs during the second half of pregnancy (2). The development of animal models of preeclampsia has proven challenging as they are required to demonstrate most of the preeclamptic symptoms such as maternal hypertension, renal dysfunction with proteinuria, endothelial dysfunction, poor trophoblast invasion, and abnormal fetal outcomes. Animal models proposed for these studies include placental hypoxia (reduced uterine perfusion pressure; RUPP), altered activity of the renin-angiotensin-aldosterone system (transgenic), impaired angiogenesis (sFLT-1 infusion), nitric oxide abnormalities (NO synthase knockout mice), immunological models (TNF-α infusion), and models of chronic hypertension (stroke prone spontaneously hypertensive rat; SHRSP) (3–5). None of these models fulfil all criteria or maternal and fetal parameters to be classified as ideal preeclampsia model. However, they all represent models of hypertensive pregnancy. The quest to understand these animal models remains attractive to researchers to understand the cause and to explore management strategies of hypertensive pregnancy.

To truly mimic the human condition, placentation in animal models should be comparable. However, none of the primate or rodent models represent completely the human placenta. Rodent models, especially rats, share hemochorial placentation features such as deep intrauterine trophoblast cell invasion and trophoblast-directed uterine spiral artery remodeling with humans (6). Moreover, they are commonly used because of easy housing conditions and short gestational length (20–22 days).

In this study, we characterized and compared the differentially expressed placental proteome of two rodent model of hypertensive pregnancy: SHRSP and a transgenic RAS-activated genetically induced preeclampsia-like model in Sprague-Dawley rats (SD-PE) (7). SHRSP are an established model of genetic predisposition to hypertension, representing the subset of chronic hypertensive pregnancy wherein there is strong maternal risk factor. Previous studies have characterized the pregnancy in SHRSP and shown abnormal placentation with deficient uterine artery remodeling and altered maternal response compared with normotensive Wistar–Kyoto (WKY) rats (8, 9). SD-PE represents the subset of preeclampsia with no predisposition of maternal hypertension (7). Sprague-Dawley female rats transgenic for the human angiotensinogen gene (*hAogen*) develop proteinuria and hypertension when mated with male rats transgenic with the human renin gene. This model is also associated with altered placentation and endothelial dysfunction (10, 11). During pregnancy, both models show common features such as high blood pressure, reduction of blood pressure before delivery, new-onset of proteinuria, endothelial dysfunction, reduced placental and fetal weight, small litter size, and reduced fetal number (7–11). As SHRSP have preexisting hypertension, they remain hypertensive throughout the pregnancy, whereas SD-PE develop hypertension after *day 10* from mating (7–11). The preeclampsia-like phenotype develops in parallel with abnormal placentation in both SD-PE and SHRSP model. In the present study, we compared the placenta of SD-PE and SHRSP rat models using proteomic techniques to explore the similarities and differences in the physiology of placentation on a molecular level.

## MATERIALS AND METHODS

### Animals

#### Model 1.

WKY (*n* = 5) and SHRSP (*n* = 5) rats were housed under controlled lighting (from 0700 to 1900 h) and temperature (21°C ± 3°C) and received a normal diet (rat and mouse No.1 maintenance diet, Special Diet Services, UK) provided ad libitum. All animal procedures were approved by the Home Office according to regulations regarding experiments with animals in the United Kingdom (Project License Number 60/4286).

#### Model 2.

Female hAogen TGR rats were mated with hRen TGR male (preeclamptic Sprague-Dawley, SD-PE, *n* = 5) and Sprague-Dawley females were mated with a Sprague-Dawley male as controls (pregnant Sprague-Dawley, SD-C, *n* = 5). All rats were held under standard conditions (average 22°C room temperature, a humidity of 55% ± 15% and 12:12-h light/dark cycle At the end of the experiment according to the European law for animal protection, rats were sacrificed under isoflurane anesthesia by decapitation. Local authorities have approved these studies (State Office of Health and Social Affairs, Berlin).

#### Both models.

Females were time mated at 12 wk of age (±4 days). Placenta were collected after 3 wk of pregnancy at gestational *day 18.5*. The method of euthanasia was terminal anesthesia using isoflurane for all the animals. All the maternal, fetal, and placental characterization of rat models is available in our previous publications (9, 11, 12) (Supplemental Fig. S1, all Supplemental material is available at https://doi.org/10.6084/m9.figshare.13490262).

**Figure 1.**
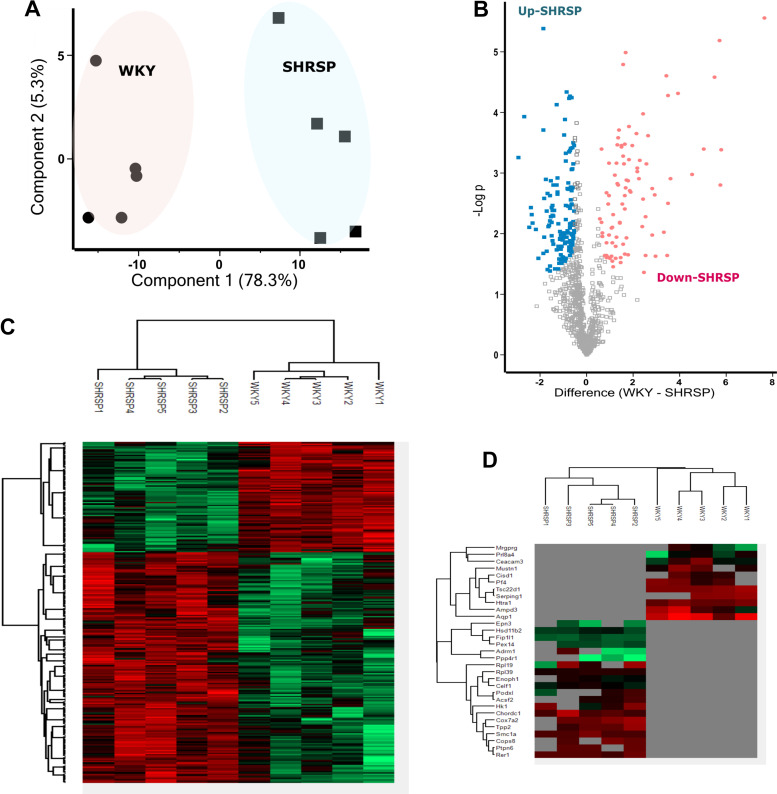
Model 1 SHRSP vs. WKY placenta proteome. *A*: principal component analysis plot of the differentially expressed protein. Percentages represent variance captured by principal component 1 and 2. Circles represent WKY and square represents SHRSP. *B*: volcano plot showing negative natural log of the *P* values plotted against the log(base2) of the change for each of the proteins quantified by label-free proteomic analysis comparing WKY and SHRSP. Proteins were downregulated (*P* < 0.05) and upregulated (*P* < 0.05) in SHRSP are colored pink and blue respectively. *C* and *D*: clustered heat map of differentially expressed protein label-free quantification (LFQ) profiles between WKY and SHRSP after *t* test analysis. Red, increased protein expression; green, low relative to the other samples; Gray, zero LFQ. SHRSP, stroke-prone spontaneously hypertensive rats; WKY, Wistar–Kyoto.

### Placental Tissue Collection

Placental tissue was harvested and dissected into two parts: mesometrial triangle and labyrinth with junctional zone. Tissues were snap frozen in liquid nitrogen and stored in −80°C until further use. In case of *model 2*, the labyrinth with junctional zone was transported to University of Glasgow in dry ice for protein extraction and proteomic analysis.

### Protein Extraction and Tryptic Digestion

Proteome analysis was performed for the labyrinth with junctional zone (here onward placenta). Five placental tissue samples per group (1 placenta per rat) were randomly selected for the study. Frozen tissues were thawed and homogenized in lysis buffer containing 1 M urea, 0.2 M thiourea, 70 mM dithiothreitol, and 0.1% octyl-β-thioglucopyranoside. The homogenate was centrifuged at 25,000 *g* for 30 min at 25°C. The supernatant was collected and total protein was estimated using Quick Start Bradford protein Assay (Bio-Rad Laboratories).

Furthermore, 100 µg protein were digested after desalting with Amicon Ultra 3 kDa MWCO (Millipore) using 50 mM ammonium bicarbonate. The protein samples were subjected to heat denaturation (80°C), reduced using dithiothreitol for 30 min at 65°C, and alkylated with iodoacetamide in the dark for 30 min at 25°C. Trypsin (Trypsin Gold, MS grade, Promega) was added in 1:20 ratio trypsin:protein (w/w) and incubated overnight at 37°C. The reaction was stopped by the addition of 2 µL of formic acid.

### NanoLC MS/MS Analysis and Quantification

LC-MS/MS was performed on an UltiMate 3000 nano-flow system (Dionex/LC Packings) connected to an LTQ Orbitrap hybrid mass spectrometer Velos FTMS (Thermo Fisher Scientific, Germany) equipped with a nano-electrospray ion source. After loading (5 µL) onto a Dionex 0.1 × 20 mm 5-µm C18 nano trap column at a flowrate of 5 µL/min in 98% water, 0.1% formic acid, and 2% acetonitrile, sample was eluted onto an Acclaim PepMap C18 nano column 75 µm × 50 cm, 2 µm 100 Å at a flow rate of 0.3 µL/min. The trap and nano flow column were maintained at 35°C. Elution was with a linear gradient of solvent A, 0.1% formic acid and acetonitrile (98:2) against solvent B, 0.1% formic acid and acetonitrile (20:80) starting at 1% B for 5 min rising to 20% at 360 min then to 45% B at 480 min. The sample was ionized in positive ion mode using a Proxeon nano spray ESI source (Thermo Fisher Scientific, Hemel, UK) and analyzed in an Orbitrap Velos FTMS (Thermo Finnigan, Bremen, Germany). The column was then washed and reequilibrated before the next injection. Ionization voltage was 2.8 kV and the capillary temperature was 250°C. The mass spectrometer was operated in data-dependent MS/MS mode scanning from 380 to 1,600 amu. The top 20 multiply charged ions were selected from each full scan for MS/MS analysis using HCD at 40% collision energy. The resolution of ions in MS1 was 60,000 and 7,500 for HCD MS2. MS raw data files were searched against the Uniprot rat nonredundant database using MaxQuant v1.6.2.1. Label free quantitation was used for analysis using MaxQuant designed algorithm (13). The statistical analysis was performed using Perseus v1.6.2.3 (14).

### Gene Ontology and Protein Interaction Network

Cytoscape v3.8.0 with STRING app was used to search and visualize for protein-protein networks of differentially expressed proteins (15, 16). Gene ontology enrichment was performed using ClueGO app for Cytoscape (17).

## RESULTS

### Model 1: SHRSP versus WKY Placenta

**Figure 2.**
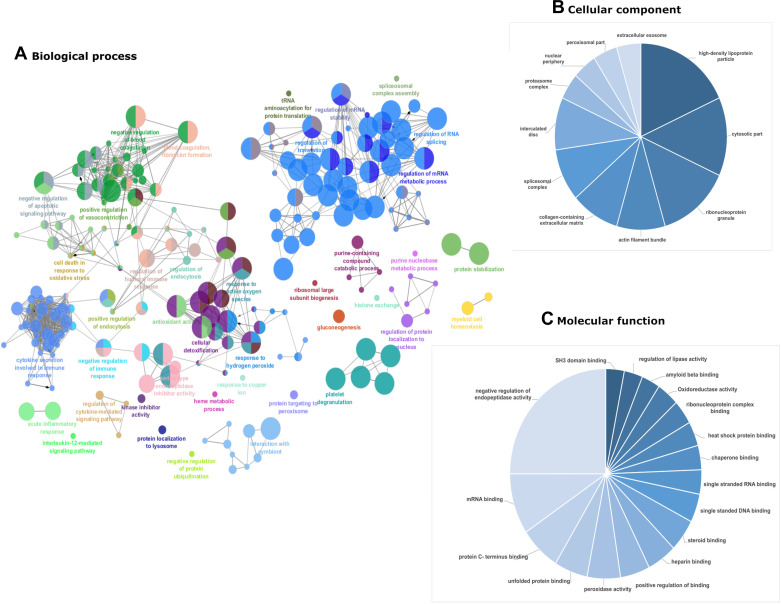
Gene ontology for *model 1* SHRSP vs. WKY placenta proteome. Enrichment of gene ontology (GO) was done using the ClueGo plugin in Cytoscape software. Figure represents the enriched GO terms for biological process (ClueGo network) (*A*), cellular component (pie chart) (*B*), and molecular function (pie chart) (*C*). In biological process enrichment network, each circle represents a node (specific GO biological process), different color symbolizes a different biological process, and size of node represents *P* value of enrichment of the GO term (smaller indicated by larger node size). The mixed color of node specifies the involvement of proteins in multiple biological processes. The connection between the two nodes (edge) indicates that the two biological process share proteins. GO analysis for all the downregulated and upregulated differentially expressed protein were also done separately (Supplemental Fig. S1). SHRSP, stroke-prone spontaneously hypertensive rats; WKY, Wistar–Kyoto.

In total, 1,058 proteins were identified after filtering for reverse identification, contaminant, valid values, proteins identified by at least two peptides or one unique peptide and 1% FDR. The label-free analysis identified 221 differentially expressed proteins with *P* < 0.05 and fold change greater than 1.5 (Supplemental Table S1). Principal component analysis (PCA) plot of the normalized (log 2 transformed) label-free quantification (LFQ) intensities for all differentially expressed proteins showed 78.3% variation at principal component (PC) 1 (Fig. 1*A*). Volcano plot and hierarchical clustering analysis grouped the differentially expressed protein into 137 upregulated and 84 downregulated proteins in SHRSP compared with WKY (Fig. 1, *B* and *C*). Hierarchical clustering also revealed 11 and 20 proteins that were detectable only in WKY or SHRSP, respectively **(**Fig. 1*D*). Gene ontology enrichment was performed with ClueGo to describe the cellular, molecular function, and biological process of differentially expressed protein in SHRSP as shown in Fig. 2. Enrichment of gene ontology of cellular components of downregulated proteins in SHRSP showed that most of these proteins belong to extracellular space, high density lipoprotein particles, extracellular organelles, collagen, and fibrinogen matrix proteins, whereas the upregulated proteins were enriched with nuclear proteins such as spliceosomal complex, cytosolic ribosome, and proteasome complex (Supplemental Fig. S2). In accordance with the cellular component enrichment, the upregulated subset of differentially expressed proteins in SHRSP was mainly enriched in molecular function terms such as RNA binding and translation factor activity (Supplemental Fig. S3). In terms of biological process enrichment, the upregulated proteins in SHRSP were from RNA splicing, regulation of post transcriptional regulation expression (Supplemental Fig. S2); whereas the downregulated proteins in SHRSP were mainly enriched in biological processes related to wound healing, coagulation and complement pathway, antioxidant activity, and negative regulation of endopeptidase activity (Supplemental Fig. S2).

### Model 2: Transgenic *hAogen* SD-PE versus Sprague-Dawley Placenta

**Figure 3.**
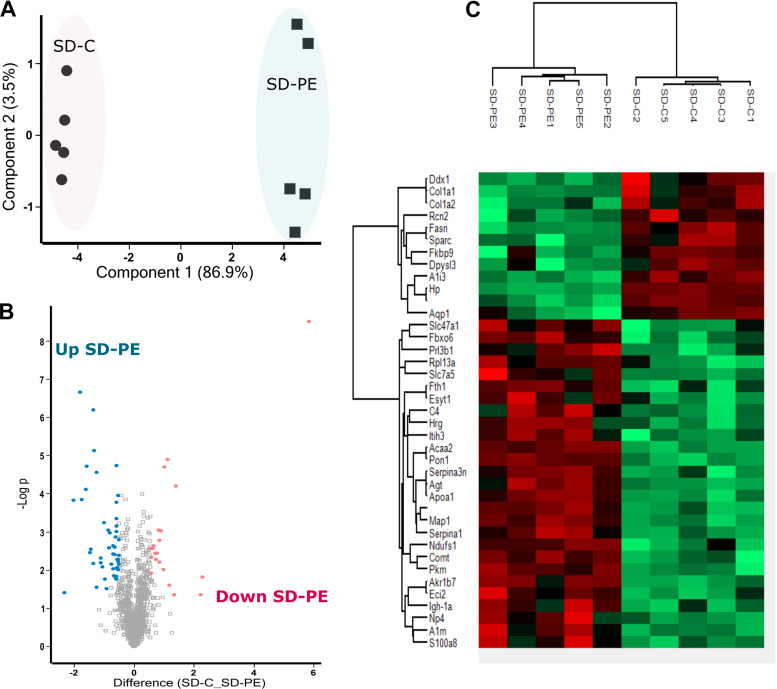
*Model 2* SD PE vs. SD control placenta proteome. *A*: principal component analysis plot of differentially expressed protein. Percentages represent variance captured by principal components 1 and 2. Circles represent SD control (SD-C) and square represents SD preeclamptic (SD-PE). *B*: volcano plot showing negative natural log of the *P* values plotted against the log(base2) of the change for each of the proteins quantified by label-free proteomic analysis comparing SD-C and SD-PE. Proteins were downregulated (*P* < 0.05) and upregulated (*P* < 0.05) in SD-PE colored pink and blue respectively. *C*: clustered heat map of differentially expressed protein label-free quantification (LFQ) profiles between SD-C and SD-PE after *t* test analysis. Red, increased protein expression; green, low relative to the other samples. SD, Sprague-Dawley.

A total 1,380 proteins were identified in *model 2* after filtering for criteria described in *Model 1: SHRSP versus WKY Placenta*. Label-free quantification identified 42 differentially expressed proteins with fold change >1.5 and *P* < 0.05 (Supplemental Table S3). PCA plot shows that these differentially expressed proteins contribute to 86.9% of variation (PC1) in SD-C and SD-PE (Fig. 3*A*). These proteins can be grouped into 13 downregulated and 29 upregulated protein in SD-PE as shown by Volcano plot and hierarchical clustering (Fig. 3, *B* and *C*). We identified two proteins (PDIA5 and S100a1) and one protein (ANK3) that were detectable only in SD-PE and SD-C, respectively.

**Figure 4.**
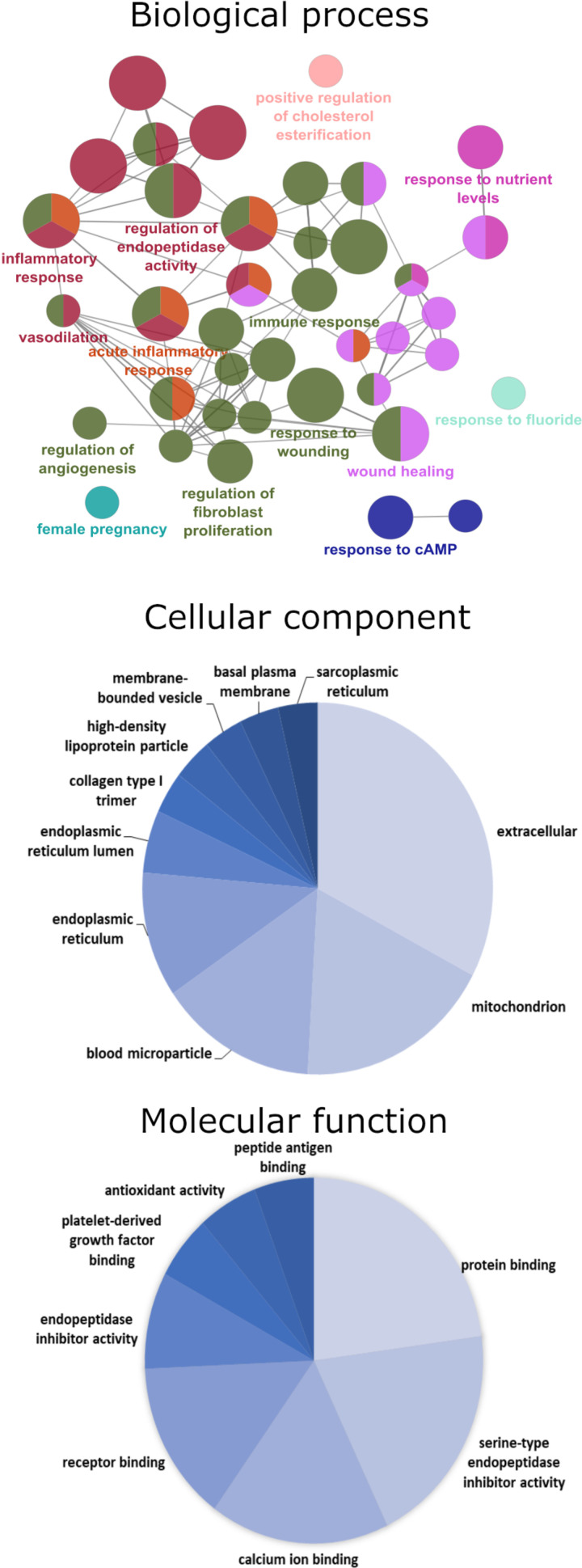
Gene ontology for *model 2* SD preeclamptic (SD PE) vs. SD control placenta proteome. Enrichment of gene ontology (GO) was done using the ClueGo plugin in Cytoscape software. GO analysis for differentially expressed protein (up- and downregulated combined) was performed for the enrichment of GO terms for biological process (*top*: ClueGo network), cellular component (*middle*: pie chart), and molecular function (*bottom*: pie chart). In biological process enrichment network, each circle represents a node (specific GO biological process), different color symbolizes a different biological process and size of node represents *P* value of enrichment of the GO term (smaller indicated by larger node size). The mixed color of node specifies the involvement of proteins in multiple biological processes. The connection between the two nodes (edge) indicates that the two biological process share proteins. SD, Sprague-Dawley.

Gene ontology enrichment for the down- and upregulated proteins separately did not show any specific pathway enriched with *P* < 0.05 and minimum number of three gene interactions. Thus, we combined the down- and upregulated proteins for the further analysis of gene ontology enrichment in ClueGo with minimum of two gene interactions. This resulted in the enrichment of cellular component terms such as extracellular space, mitochondria, and endoplasmic reticulum, and molecular function enrichment terms such as protein binding, serine-type endopeptidase inhibitor activity, calcium ion binding, receptor binding, and antioxidant activity (Fig. 4).

## DISCUSSION

We conducted an indepth analysis of the placental proteome of two rodent models of hypertensive pregnancy that provides the basis for more targeted translational research.

### SHRSP versus WKY Placenta

The major cluster of proteins with 1 downregulated, 26 upregulated, and 4 proteins detected only in SHRSP belongs to RNA metabolism such as spliceosome, mRNA splicing, mRNA transport, and stabilization (Supplemental Fig. S3). The most abundant RNA binding family expressed are heterogeneous nuclear ribonucleoproteins (HNRNPs) such as HNRNPA2B, HNRNPA3, HNRNPD, HNRNPF, HNRNPH1, and HNRNPL. Some of these RNA binding proteins are involved in hypoxia response (HNRNPD, ELAVL1, HNRNPA2B1, KHSRP, and CELF1) (18) and posttranscriptional regulation of the antiapoptotic BCL2 pathway (HNRNPD, HNRNPF, PTBP1, ELAVL1, HNRNPA2B1, and SF3B4) (19). Serine/threonine kinase receptor associated protein (STRAP) was observed to be particularly highly expressed (FC 18.7, *P* < 0.0001) in this cluster. STRAP has chaperoning function of the SMN complex involved in splicesomal snRNP assembly (20) and is also implicated in negative regulation of the TGF-β signaling pathway via binding to TGF-β receptor (21).

In SHRSP, the antioxidant activity was enriched in downregulated proteins whereas the hydrogen peroxide metabolic process was enriched in the upregulated proteins (Fig. 2*A*). Oxidative stress is one of the features of hypertensive pregnancy and placenta is unarguably one of the free radical contributors. The mitochondrial superoxide formed is rapidly catalyzed by SOD2 to hydrogen peroxide which is further detoxified using catalase, glutathione peroxidases (GPX), or peroxidases (PRDX). Our data indicate upregulation of mitochondrial SOD2 and downregulation of main detoxification pathway protein viz. catalase and GPX1 in SHRSP, which might lead to mitochondrial accumulation of H_2_O_2_. However, our data also suggest that upregulation of mitochondrial PRDX3 and PRDX5 in SHRSP which might help in reducing the toxicity of H_2_O_2_ accumulation. In preeclampsia, oxidative stress is mostly a result of hypoxia or ischemia-reperfusion injury. Indeed, we found interaction of hypoxia specific proteins such as downregulated proteins serotransferrin, SERPINA1, haptoglobin, α-B crystallin, and aquaporin and upregulated proteins adenosyl homocysteinase, SOD2, and mitochondrial 60 kDa heat shock protein (Supplemental Fig. S2). α-B Crystallin also promotes angiogenesis via modulating VEGFA, negatively regulates apoptosis, and protects cells from stress-induced damages as a small heat shock protein/chaperone (22).

Amine oxidase copper-containing 1 (AOC-1, also known as diamine oxidase; DAO) is one of the lowest expressed proteins (224-fold, *P* < 0.001) in SHRSP. AOC-1 is a histamine degrading enzyme which is highly expressed in the placenta (more than 100-fold) during normal pregnancy (23). Low AOC-1 activity and balance between histamine and AOC-1 is implicated in high risk pregnancies and preeclampsia (23, 24). We have validated these results in SHRSP and WKY placenta with immunohistochemistry and Western blot (Supplemental Fig. S4)

### Transgenic *hAogen* SD-PE versus Sprague-Dawley Placenta

Biological process gene annotation enrichment pointed toward regulation of endopeptidase activity, inflammatory response, wound healing, and immune response (Fig. 4). As SD-PE is a model of transgenic human angiotensinogen (AGT), it was not surprising to find the fourfold increased expression of AGT in SD-PE and its involvement in most of gene ontology terms such as vasodilation, angiogenesis, inflammatory response, and pregnancy. We found that AGT interacts with other differentially expressed proteins viz. aquaporin-1 (AQP1), collagen α-1(I) chain (COL1A1), and kininogen (KNG). T-kininogen 1 and 2 are precursors of bradykinin only found in rat and show 2.6- and 3-fold increased expression in SD-PE; T-kininogens are proinflammatory acute phase proteins (25). Another protein that is associated with angiotensin-mediated inflammatory response and preeclampsia is catechol O-methyltransferase (COMT), which was found to be increased 1.5-fold in SD-PE. COMT deficiency and polymorphism is implicated in the pathophysiology of hypertensive pregnancy disorders and Ang-II hypersensitivity (26, 27). Increased expression of COMT was previously observed in the renal vascular wall of the double transgenic human renin and angiotensinogen rats and entacapone (COMT inhibitor) was shown to provide protection against Ang-II induced renal damage (28).

### SHRSP versus Transgenic SD-PE

In the current study, we compared the placental proteome between a transgenic and a genetically predisposed hypertensive rat model. A direct proteome comparison of SD-PE to SHRSP led to false positive identification of more than 900 differentially expressed proteins (data not shown) that mainly consisted of strain-specific differences. Thus, we compared each model to its respective control rat and further analyzed the differentially expressed proteins from each group. Each model shows only certain features of preeclampsia of which some were common between the models. In SHRSP placenta, oxidative stress, hypoxia, and inflammation were observed to be prominent which may derive from systemic vasculature (29), whereas SD-PE being transgenic for components of the renin-angiotensin-aldosterone system (RAAS) (7) showed pathways related to angiotensin and angiogenesis. We are confident that our analysis is valid as we were able to demonstrate differential regulation of some of the key pathogenetic principles including RAAS activation and oxidative stress in these models. In line with our proteomic data, Scott et al. (30), through transcriptomics of uterine arteries, show an elevated immune response, and increased production of reactive oxygen species and downstream effectors of the RAAS in pregnant SHRSP.

On comparing differentially expressed proteins from SHRSP and SD-PE, 15 proteins were found common among them. As shown in Table 1, 10 of these proteins were either up- or downregulated in SHRSP or SD-PE. Four proteins, viz α-1-inhibitor 3, AQP-1, RT1 class I histocompatibility antigen AA α chain and haptoglobin were downregulated, whereas 3-ketoacyl-CoA thiolase was upregulated both in SHRSP and SD-PE.

**Table 1.** List of differentially expressed proteins common to SHRSP and SD-PE

Proteins	SHRSP	SD-PE	Function
Serum paraoxonase (PON1)	↓ (0.2***)	↑ (2.6****)	Antioxidant; anti-inflammatory; positive regulation to cholesterol efflux
α-1-inhibitor 3 (A1I3)	↓ (0.5**)	↓ (0.5****)	Acute phase protein; serine-type endopeptidase inhibitor activity
α-1-macroglobulin (A1M)	↓ (0.4****)	↑ (1.6***)
Serine protease inhibitor A3N (SERPINA3N)	↓ (0.2**)	↑ (3.3****)
α-1-antiproteinase (SERPINA1)	↓ (0.3***)	↑ (1.5****)
Inter-α-trypsin inhibitor heavy chain H3 (ITIH3)	↓ (0.2***)	↑ (1.8***)
Ferritin heavy chain (FTH1)	↓ (0.5***)	↑ (1.5***)	Cellular iron homeostasis; immune response; negative regulation of cell population proliferation
Large neutral amino acids transporter small subunit 1 (SLC7A5)	↓ (0.4**)	↑ (1.5**)	Amino acid transmembrane transporter activity; leukocyte migration
Aquaporin-1 (AQP1)	↓(nd)	↓ (0.7**)	Transport of ammonium, bicarbonate, CO_2_, and water; cellular hyperosmotic response; response to hypoxia, inorganic substance, salt stress, drug; positive regulation of angiogenesis, fibroblast proliferation; negative regulation to apoptotic process
RT1 class I histocompatibility antigen, AA α chain (HLA-A)	↓ (0.1**)	↓ (0.5****)	Antigen processing and presentation of peptide antigen via MHC class I; positive regulation of T cell-mediated cytotoxicity
Apolipoprotein A-I (APOA1)	↓ (0.5**)	↑ (1.5****)	Cholesterol homeostasis; negative regulation of inflammatory response, interleukin-1 β secretion, TNF-mediated signaling pathway
Haptoglobin (HP)	↓ (0.2***)	↓ (0.01****)	Acute phase protein; antioxidant activity
Ig γ-2B chain C region (IGH-1A)	↓ (0.1****)	↑ (2.8**)	Antigen binding; complement activation; B-cell receptor signaling pathway
Fatty acid synthase (FASN)	↑ (3.1**)	↓ (0.4****)	Acetyl-CoA metabolic process; differentiation of monocyte, neutrophil, osteoblast
3-ketoacyl-CoA thiolase, mitochondrial (ACAA2)	↑ (2.7***)	↑ (3.5****)	Fatty acid β-oxidation; cellular response to hypoxia; negative regulation of mitochondrial membrane permeability involved in apoptotic process

Bracket in column 2 and 3 represents (fold change, *P* value); fold change in column 2 is comparison SHRSP/WKY, column 3 is comparison SD-PE/SD-C, and nd is not detected in strain. Symbol represents: ↓, downregulation; ↑, up regulation; **P < 0.01, ***P < 0.001, ****P < 0.0001. SD-C, SD control; SD-PE, SD preeclamptic; SD, Sprague-Dawley; SHRSP, stroke-prone spontaneously hypertensive rats; WKY, Wistar–Kyoto.

As hypertension is the common theme in both models, certain differentially expressed proteins in both models are regulated by high blood pressure. Inflammation is implicated in the development of hypertension and preeclampsia, either as a primary or secondary event. In response to inflammation, the concentration of acute phase protein in plasma either increase or decrease. Haptoglobin (HP) is an acute phase protein mainly secreted by the liver and was found to be downregulated in both SHRSP and SD-PE. HP is known to play antioxidant, proangiogenic, and anti-inflammatory roles (31). Our previous study shows that HP is downregulated in placenta and plasma of women with preeclampsia (32). α-1 Inhibitor III (α1I3), a negative acute phase protein found only in rats (but shares similarity to α-2 macroglobulin in humans) and strongly downregulated during acute and chronic inflammations (33) was also downregulated in both models; whereas α-1 macroglobulin was downregulated in SHRSP and upregulated in SD-PE. Acute phase proteins such as serine protease inhibitors (SERPIN) are known to be involved in angiogenesis, coagulation, fibrinolysis, cell migration, and inflammation (34). In SHRSP, SERPINA3N, SERPINA1, and inter-α-trypsin inhibitor heavy chain H3 (ITIH3) were downregulated whereas these were upregulated in SD-PE. The regulation of acute phase proteins observed in our data is mostly opposite between SD-PE and SHRSP. Initiation, continuation, and intensity of inflammation in chronic hypertensive SHRSP might differ from the acute inflammation during gestation seen in SD-PE.

One of the major differences between these models is the deficient trophoblast invasion of uterine spiral artery observed in SHRSP and the contrasting deeper trophoblast invasion found in transgenic preeclampsia rat model (9, 35, 36). Anti-inflammatory proteins such as PON-1 (removes ox-LDL that inhibit trophoblast invasion) (37) and apolipoprotein A1 (protective role in trophoblast invasion) (38) were found to be upregulated in SD-PE whereas downregulated in SHRSP. Suppression of LAT-1 (Slc7a5), a sodium-dependent large neutral amino acid transporter, leads to inhibition of trophoblast invasion (39). In our data, LAT-1 was found to be upregulated in SD-PE and downregulated in SHRSP. However, AQP-1 was found to be downregulated 1.5-fold in SD-PE whereas in case of SHRSP it was not detectable and found to be highly expressed in WKY. AQP1 is a water channel, however it is known to play a significant role in angiogenesis, mostly because of its expression in the placental vasculature (40, 41). Studies in knockout *Aqp1*^−/^ (loss of maternal allele) and *Aqp1*^−/−^ mice show that AQP1 is expressed maternally in the placenta and its deficiency leads to placental abnormalities (42) and also altered blood vessel structure and increased syncytiotrophoblast nodules (43). Primary cell cultures of aortic endothelia from AQP1 null mice show impaired cell migration and angiogenesis (44). Interestingly, transgenic mice overexpressing renal rat angiotensinogen showed angiotensinogen-mediated downregulation of AQP1 via nuclear factor erythroid 2-related factor 2-heme oxygenase-1 pathway (45).

Interaction between HLA class 1 and uterine natural killer (uNK) cells plays an important role in trophoblast invasion. In rats, NK cells are inhibited by RT HLA class1.A (46). Our data showed decreased RT HLA class 1 in placenta of both models, thus complementing the previous studies showing increased uNK cells in placenta of both SHRSP and SD-PE (10, 47).

One of the limitations of this study is that animals were housed separately, however, sample preparation and mass spectrometry analysis were performed together to maintain consistency in the data. The proteins identified and represented in our data (few 1,000) is just subset of the total proteome. The complete proteome identification is solely depended on the sample preparation, enrichment, fractionation, and capability of mass spectrometry instrument. Differences in experimental design and data handling makes data comparison challenging in nontargeted proteomics. We and others have therefore suggested development of standards for preclinical and clinical proteomics (48, 49). However, untargeted approach also has merits, for example, testing pharmaceutical interventions, such data can help to identify the model that is best suited for such experiments (50). This is the first study to report the difference in the placental proteome of two hypertensive pregnancy models. Despite sharing common clinical features of hypertensive pregnancy, these models are diverse in their placental pathophysiology. This is similar to the situation in humans where the broad clinical definition of preeclampsia is met by women with different risk factors, course of disease and likely, pathophysiology. This is evident in a recent metaanalysis of 23 references (year 2007–2020) on human placenta proteomics studies that showed only 16.9% of differentially expressed proteins identified are common in two or more studies (51).

With markedly different molecular features between models it is also likely that the pathophysiology of preeclampsia and chronic hypertension pregnancy is not exclusively driven by the placenta. There is no ideal animal model for preeclampsia or hypertensive pregnancy, however, molecular (proteomic) characterization can help to describe features of models that will inform their use for comparative studies with human placenta and for preventative and therapeutic studies.

## GRANTS

This study was funded by the Academy of Medical Sciences-Newton International Fellowship Grant NIF004\1010 (to S.M.). C.D. is supported by a British Heart Foundation Center of Research Excellence Award (RE/18/6/34217).

## DISCLOSURES

No conflicts of interest, financial or otherwise, are declared by the authors.

## AUTHOR CONTRIBUTIONS

S.M., H.S., R.D., and C.D. conceived and designed research; S.M., H.S., F.H., E.C., A.F., and W.M. performed experiments; S.M. analyzed data; S.M., F.H., W.M., R.D., and C.D. interpreted results of experiments; S.M. prepared figures; S.M. drafted manuscript; S.M., H.S., F.H., W.M., R.D., and C.D. edited and revised manuscript; S.M., H.S., F.H., E.C., A.F., W.M., R.D., and C.D. approved final version of manuscript.
